# Co-evolutionary Dynamics of Collective Action with Signaling for a Quorum

**DOI:** 10.1371/journal.pcbi.1004101

**Published:** 2015-02-23

**Authors:** Jorge M. Pacheco, Vítor V. Vasconcelos, Francisco C. Santos, Brian Skyrms

**Affiliations:** 1 Centro de Biologia Molecular e Ambiental, Universidade do Minho, Braga, Portugal; 2 Departamento de Matemática e Aplicações, Universidade do Minho, Braga, Portugal; 3 ATP-Group, CMAF, Instituto para a Investigação Interdisciplinar, Lisboa, Portugal; 4 Centro de Física da Universidade do Minho, Braga, Portugal; 5 INESC-ID and Instituto Superior Técnico, Universidade de Lisboa, IST-Taguspark, Porto Salvo, Portugal; 6 Logic and Philosophy of Science, School of Social Sciences, University of California at Irvine, Irvine, California, United States of America; University of Washington, UNITED STATES

## Abstract

Collective signaling for a quorum is found in a wide range of organisms that face collective action problems whose successful solution requires the participation of some quorum of the individuals present. These range from humans, to social insects, to bacteria. The mechanisms involved, the quorum required, and the size of the group may vary. Here we address the general question of the evolution of collective signaling at a high level of abstraction. We investigate the evolutionary dynamics of a population engaging in a signaling *N*-person game theoretic model. Parameter settings allow for loners and cheaters, and for costly or costless signals. We find a rich dynamics, showing how natural selection, operating on a population of individuals endowed with the simplest strategies, is able to evolve a costly signaling system that allows individuals to respond appropriately to different states of Nature. Signaling robustly promotes cooperative collective action, in particular when coordinated action is most needed and difficult to achieve. Two different signaling systems may emerge depending on *Nature*’s most prevalent states.

## Author Summary

From humans to social insects and bacteria, decision-making is often influenced by some form of collective signaling, be it quorum, information exchange, pledges or announcements. Here we investigate how such *signaling* systems evolve when collective action entails a public good, and how meanings co-evolve with individual choices, given Nature’s most prevalent states. We find a rich scenario, showing how natural selection is able to evolve a costly quorum signaling system that allows individuals to coordinate their action so as to provide the appropriate response to different states of Nature. We show that signaling robustly and selectively promotes cooperative collective action when coordinated action is most needed. In light of our results, and despite the complexity that collective action relying on quorum signaling may entail, it is not so surprising how signaling is a ubiquitous property of the living world.

## Introduction

Animal collectives are known to exhibit a significant diversity of decision-making systems, most of which require some form of *counting* [1–6]. This information is gathered by means of individuals’ sensory apparatus, typically subjected to a variety of confounding factors, such as environmental noise (even in the form of deceitful information). These factors can be counteracted, in some cases, by resorting to additional social information. Such instantiations of collective action, which accrue to individuals of species exhibiting a huge variety of sensory and social hardware [7], require a high level of abstraction whenever one wants to address them in a unified way. Here we shall address the general question of how did individuals evolve mechanisms that allow them to convey a meaning to external (environmental and/or social) information in order to perform adequately as a collective. Thus, from bacteria to Humans, we shall assume that groups of individuals may face the following ubiquitous collective action problem: (*i*) a certain proportion, but not necessarily all, of the group must participate in order to produce a common good; (*ii*) participation has a cost, so participating in an unsuccessful project entails a new loss; (*iii*) the problem is sometimes present and sometimes absent. If the common good is successfully created but some did not contribute to its creation, there are two possibilities: the shirkers may either share the benefits (acting as cheaters or defectors) or be excluded (loners).

Species that face such problems are well-served by a signaling system that indicates whether (*i*) the collective action problem is present and (*ii*) there will be enough participants to successfully produce the common good (quorum sensing). Even in the simplest organisms [8–22] it has been recently found that quorum signaling systems are ubiquitous, revealing the existence of sophisticated information exchange mechanisms by which simple organisms reach consensus involving different numbers of participants with varying costs and benefits resulting from initiating collective action. Despite these differences in detail, at a high level of abstraction, the essentials can be captured in a signaling *N*-person game theoretic model.

We provide such a model and analyze the evolutionary dynamics of a population of individuals. Parameter settings allow for loners and cheaters, and allow for costly or costless signals. We find a rich dynamics, with surprising differences from what might be expected by equilibrium stability [23] analysis. We analyze the evolutionary dynamics between cooperators and shirkers, and in all cases show how natural selection, operating on a population of individuals endowed with the simplest strategies, is able to evolve a signaling system that mimics what is observed in nature. We find that signaling generally and robustly promotes cooperative collective action, even when shirkers act as cheaters and even when signaling strategies are rare, since they protect cooperation by forming a barrier against the invasion of cheaters.

We shall assume that Nature may choose between two different states, representing both adverse (state α) and favorable (state β) conditions for a group of individuals, allowing for (minimal) environmental variation. We assume that state α occurs with probability *λ* whereas β occurs with probability 1-*λ*. Examples of this sort abound across species. Among humans, groups of individuals may recognize that one of the states of Nature requires collective cooperative action (say, under the new threat of global warming), as opposed to the other, where collective cooperative action may be dispensed. By observing the state of Nature, an individual may provide cues of some sort (signals, in the form of visual cues, pledges or announcements), being possible that individuals signal differently (or not) the different states of Nature. The aggregated information will influence the collective outcome of the group [3,16,24–28]. For certain bacterial micro-organisms, the two states of Nature considered can be associated with *Starvation* (α) and *Abundance* (β). Under *Abundance* (small values of λ), bacteria typically opt for reproducing individually, in which case no collective action is required. Under *Starvation* (large values of λ), cooperation may become necessary for survival; cooperation is costly, but collective action entails a public good [14].

## Results/Discussion

Most models considered to date generally assume that a signaling system exists and performs the right function [12,15,29–37]. In this work, we are mostly interested in answering the question: How does a signaling system evolve, and under which conditions? Consequently, we assign no *a priori* meaning to the signals σ. Each individual adopts a strategy of the form (σ_α_ σ_β_ | A_0_ A_1_), where σ_α_ is the signal emitted by the individual when Nature chooses α and σ_β_ the signal emitted under β. We assume the simplest scenario of two signals (σ = 0 and σ = 1) such that σ = 0 is costless—and may be associated with the absence of a signal—whereas signal σ = 1 implies a cost (*c*_*S*_) to the emitter. Individuals interact in groups facing a *N*-person coordination dilemma [38], a public goods problem in which a coordination threshold (*M*) less or equal than the total group size (*N*) is required to produce benefits, with increasing participation leading to increasing productivity (see Methods). Individuals may react differently depending on the signal adopted by the majority: We shall adopt a majority rule here, without loss of generality; indeed, any other form of *counting* or *quorum sensing* may replace the majority rule adopted here. The values of A_i_ encode the behavior of the individual when the majority adopts signal “0” (A_0_) and signal “1” (A_1_). Whenever A_i_ = 1 individuals opt for Cooperation (***C***); if A_i_ = 0 individuals refuse to do so, acting as shirkers. Shirkers are either Defectors (***D***s), in which case they forego the cost while enjoying a share of the public good, or Loners (***L***s), abstaining from contributing to the public good and foregoing the benefits from collective action. Irrespective of whether the competition is between ***C***s and ***D***s or between ***C***s and ***L***s, we show (see S1 Text) how the present model provides the expected solutions in the two extreme cases, where no environmental variation occurs: *i)* When Nature only chooses α (*λ* = 1.0) or *ii)* when Nature only chooses β (*λ* = 0.0).

In the former case, collective action entails a public good, and the group would be better off by cooperating. However, given that a minimum number of contributions *M*≤*N* is needed to secure a collective benefit, there is always an incentive to free ride, both because cooperators may pay a cost in vain and also because free-riding may also entail a benefit for defectors at no cost [38]. If individuals cooperate, and given that they never experience β, the best case scenario will translate into cooperation in the absence of any (costly) signal. In other words, the best case scenario corresponds to the population spending all of its time in configurations of the type (0*|1*), where the placeholder “*” means here any value. Indeed, in S1 Text we show that, for *λ* = 1.0, the strategies (00|10), (00|11), (01|10) and (01|11) take 25% of the total time each.

Whenever Nature only chooses β (*λ* = 0.0), given the alternatives of emitting a costly signal (signal 1) or emitting no signal (signal 0), and given the fact that collective action is of no use in this case, individuals will be better off emitting no signal. For the same reason, they should *defect* under majority of “signal 0”. In these conditions, what they do under “signal 1” should be irrelevant, given the fact that selection cannot operate on this particular aspect of individuals’ strategy. In other words, the best case scenario corresponds to the population spending all of its time in configurations of the type (*0|0*). The results shown in S1 Text fully corroborate this picture.

In the general case (0 < *λ* < 1), if a signaling system evolves and is at work, the fact that one of the signals is costly means that individuals should employ their portfolio of signals to find a means of discriminating between the states of Nature α and β. This naturally requires that meanings co-evolve with the emergence of a signaling system. Strategies that correspond to such signaling systems are therefore strategies (10|01) and (01|10).

The former corresponds to situations in which the costly signal (“1”) is used to signal the need for cooperation under α, leading to a cooperative reaction from players that respond cooperatively to a majority of such signals. In latter case, the cheaper signal (“0”) is the one used to effect cooperation.

**Fig 1:**
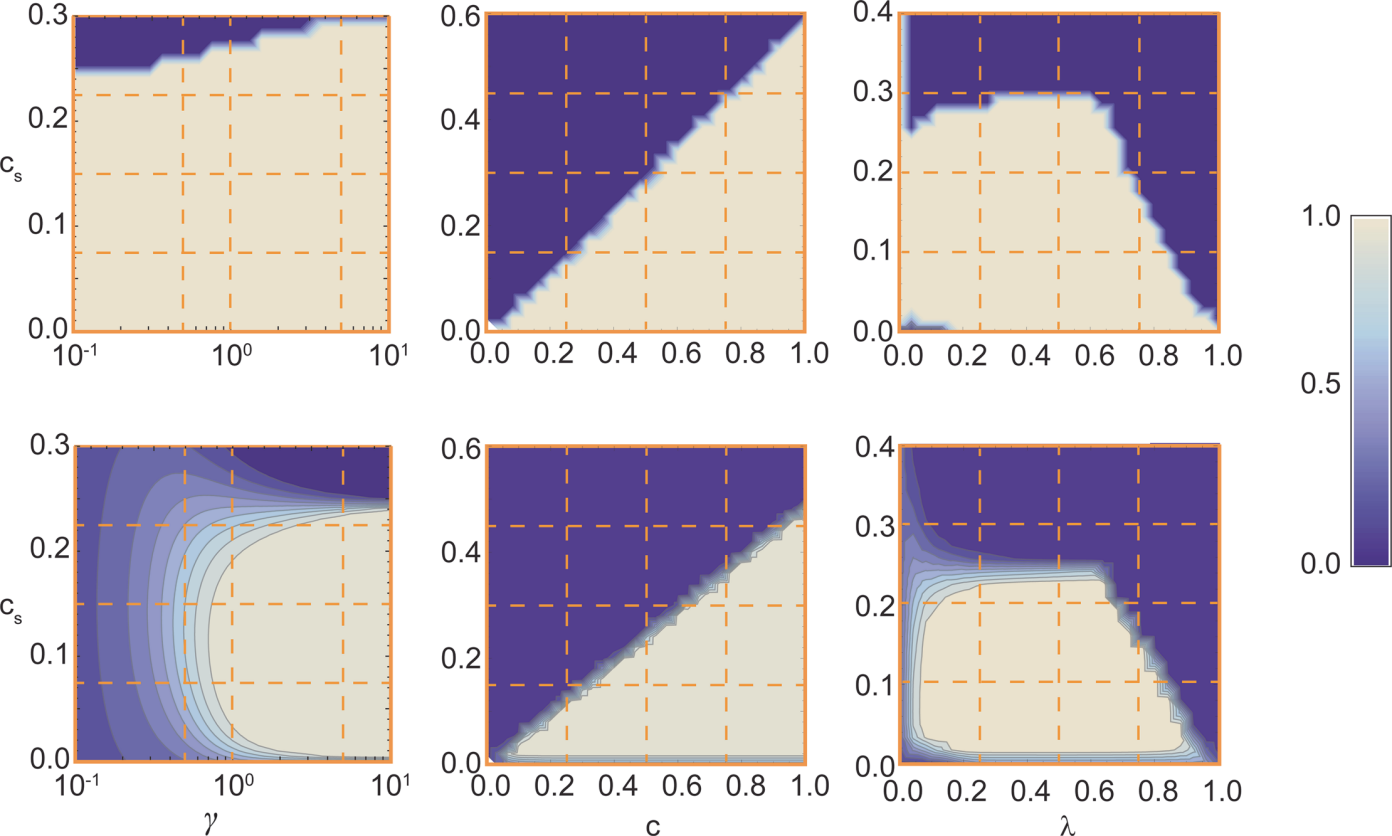
Evolutionary robustness and Prevalence of signaling strategy (10|01). The **top** contour plots indicate the regions of model parameters in which the signaling strategy (10|01) is Evolutionary Robust (ERS) (bright areas). To this end we vary the selection pressure *γ* and the *cost of signaling c*_*S*_ (left panel); *the cost of cooperation c* and *c*_*S*_ (center panel) and the probability that Nature chooses α (λ) and *c*_*S*_. The **bottom** panels show, for the same parameter space, the prevalence of the population in a configuration in which all individuals adopt the signaling strategy (10|01). The remaining parameters (and also those plotted whenever not varied) are *Z* = 100, *N* = 9, *M* = 5, *c* = 0.5, *γ =* 5, *F* = 10, *λ* = 0.5. A signaling system emerges for a wide range of values of the model parameters. Comparison of top and bottom panels shows the existence of parameter regions in which (10|01) is an ERS and yet the population almost never adopts this strategy and, conversely, there are regions in which (10|01) is not an ERS and yet the population spends approximately 30% of its time in configuration comprising only this strategy (see main text for details). Note, in particular, that signaling systems emerge more robustly under strong selection, a scenario that is very likely to occur in many cases under α conditions (those that entail a public good). Moreover, for some species the enhancement factor *F* = 10 possibly constitutes an underestimate given that, under α, survival may be at stake (as is the case in, e.g., many bacterial species), and successful cooperation likely leads to higher benefits. Nonetheless, a signaling system emerges for a wide range of values of *λ*, provided that *c*_*S*_ *< c/*2.

Our results show that, irrespective of the values of 0<λ<1 and game parameters chosen, at least one of these two signaling strategies prevails in the population, with a larger predominance of strategy (10|01), in which costly signals are used in situations where cooperation is required. As an example, in Fig. 1 (lower panels) we show the prevalence of strategy (10|01)—that is, the fraction of time the population spends in a configuration in which all individuals adopt such signaling strategy—as a function of ***a***) selection pressure *γ* and the *cost of signaling c*_*S*_; ***b***) *cost of cooperation c* and *c*_*S*_ ***c***) probability *λ* of Nature choosing α and *c*_*S*_. These results (see also figures below) were obtained when shirkers act as Defectors, but analogous results are naturally obtained when shirkers act as Loners (see S1 Text for details). Indeed, since Loners, like cheaters, abstain from contributing to the public good but, unlike cheaters, forego the benefits from collective action, the disadvantage of cooperators is less pronounced in this case, which facilitates the emergence of collective signaling.

Clearly, this signaling system manages to emerge for a wide range of values of the model parameters; it is noteworthy that it also emerges more robustly under strong selection (large *γ*), a scenario that is very likely to occur under the state of Nature α. Moreover, for this strategy to dominate, the cost of signaling must be smaller than the cost of cooperation (here *c*_*S*_ < *c/*2), in agreement with what is known from the empirical literature [39].

We may also compute the set of *Evolutionary Robust Strategies* (ERS) [40,41], defined as strategies for which natural selection opposes the fixation of a mutant adopting any other strategy (see Methods for details). In particular, comparison between the lower and upper panels of Fig. 1, where we display the regions of parameter space where the strategy (10|01) is an ERS, shows that being robust against fixation does not necessarily warrant a high prevalence of this strategy in the population. Indeed, we find regions where (10|01) is an ERS with 0% prevalence and regions where it is *not* an ERS and, despite this fact, its prevalence can be significant among all 16 possible strategies (see below). We find similar predictions with other stability or robustness measures (see Methods).

**Fig 2:**
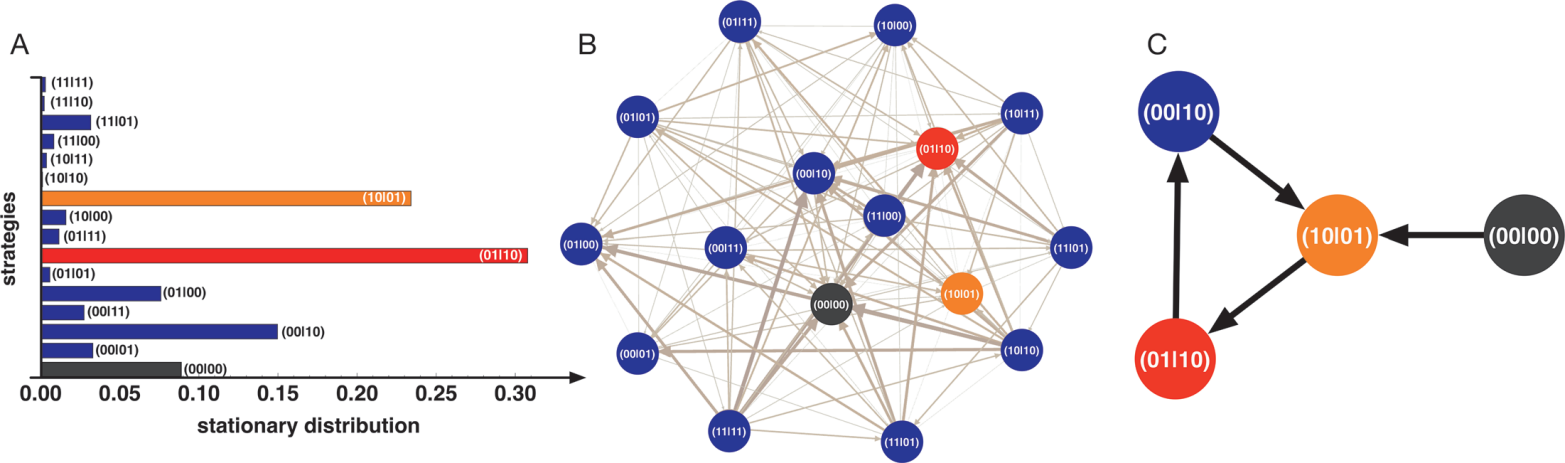
Dynamics. **A)** The bar plot shows the fraction of time the population spends in each monomorphic configuration of every strategy (σ_α_ σ_β_ | A_0_ A_1_) (see main text for details). **B)** The graph represents all strategies, one at each node, with edges representing transitions between strategies above neutral drift. For the parameter values chosen (*Z* = 100, *N* = 9, *M* = 5, *c* = 0.3, *c*_*s*_ = 0.06, *γ = 0*.5, *F* = 10, *λ* = 0.8), no strategy is Evolutionary Robust (ERS). **C)** We show the graph resulting from studying the dynamics in the subspace of strategies including the 4 most abundant in **A**, highlighting a 3 strategy loop which often occurs for large values of *λ*. When the state of Nature α occurs more often than β (here *λ* = 0.8), (10|01) can be invaded by its complementary discriminative strategy—(01|10)—benefiting from cheaper signals to achieve the same goal of coordinating collective action. (01|10), in turn, can be invaded by (00|10), thus saving the cost of signaling under *B*. This, in turn, can be invaded again by (10|01) closing a loop in probability space that precludes the emergence of any ERS. Note further, that despite not being the most prevalent strategy, (10|01) plays a crucial role, as it is the only one of those strategies in the loop that can invade (00|00).

As one approaches the regions where the signaling strategy (10|01) no longer takes over the entire population, it is illuminating to analyze in more detail the profile of the stationary distribution, in particular when the probability (λ) of an unfavorable state of nature (state α) is significant. This is shown in Fig. 2 for a representative case where both signaling strategies remain, overall, the most prevalent, despite no longer taking 100% of the time. The bar plot in Fig. 2A depicts the prevalence of each strategy in situations in which Nature chooses α with probability *λ* = 0.8.

In Fig. 2B and C we plot directed graphs in which each node corresponds to one of the 16 possible monomorphic states and respective strategies. The links (represented by arrows pointing from strategy *V* to strategy *U*) indicate those transitions favored by natural selection, i.e., those elements of the transition matrix *T* (see Methods) for which *ρ_V,U_* > *ρ_N_* ≡ 1 / *Z*. In other words, we show the paths along which mutants of a strategy located at the end of a link are advantageous regarding invasion of a population of individuals adopting the strategy corresponding to the origin of the link. Those strategies corresponding to nodes without outgoing links are, by definition, ERS thus rendering a robustness analysis, in principle, a trivial visual exercise.

Fig. 2 shows that whenever Nature predominantly chooses α, as it is the case, the complementary signaling strategy, (01|10)—a discriminative strategy in which individuals react cooperatively in the absence of a costly signal, and defect whenever a costly signal is present—may also emerge and, in some cases, prevail. In other words, individuals may resort to a collective analog of a cheaper signaling system (involving a secret, yet collective, handshake [8,9]) to coordinate in case of α (most abundant scenario), whereas in case of β they will employ the costly signal “1”. In this way, costly signals are used much less than whenever individuals coordinate into (10|01). Nonetheless, this strategy is rarely an ERS, as it is often invaded by other strategies, contrary to (10|01) which is robust against invasion in most of the parameter space (see Fig. 2), even when it does not emerge as the most frequent strategy. In fact, in many situations, none of the signaling strategies (or any other) is an ERS, as exemplified in Fig. 2, which fosters endless evolutionary cycles of invasion and fixation.

The loop shown in Fig. 2C provides a clear intuitive explanation for this fact, showing the transitions that link the four most abundant strategies in this scenario and which, as the bar plot in Fig. 2A shows, include the two signaling strategies. Clearly, when α occurs more often than β (here λ = 0.8), (10|01) can be invaded by its complementary discriminative strategy—(01|10)—benefiting from cheaper signals to achieve the same goal of coordinating collective action. Strategy (01|10), in turn, can be invaded by (00|10), thus saving the cost of signaling under α. This, in turn, can be invaded again by (10|01) closing a loop in probability space that precludes the emergence of any ERS. Importantly, and despite not being the most prevalent strategy, (10|01) plays a crucial role, as it is the only one of those strategies in the loop that can invade (00|00).

The absence of robust strategies may naturally depend on the cost of signaling. As we decrease the ratio *c_S_/c*, there will be a critical value below which both signaling strategies may become ERS with comparable frequencies, showing that the path to the emergence of a signaling system is not unique.

**Fig 3:**
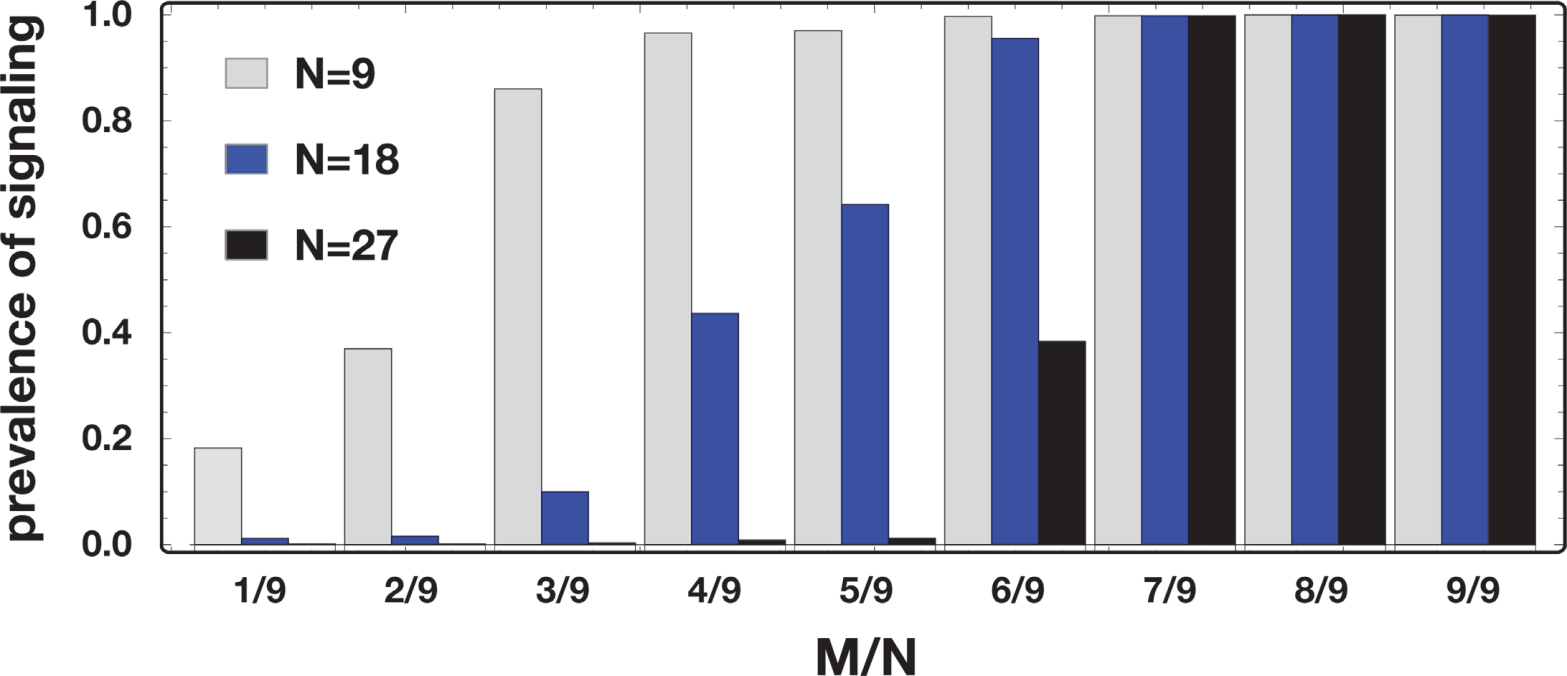
Prevalence of signaling strategies (10|01) and (01|10). We consider three different group sizes, *N* = 9 (gray bars), *N* = 18 (blue bars) and *N* = 27 (black bars), and different values of the coordination threshold *M*. Signaling strategies prevail in those situations in which coordination is harder to achieve (large *M*). Furthermore, larger groups must be subjected to stronger requirements such that signaling emerges and the public good is achieved. Other parameters: *Z* = 100, *c* = 0.5, *c*_*s*_ = 0.2, *γ* = 2, *F* = 10, *λ* = 0.5.

Moreover, the dynamical scenario discussed above remains robust for a wide interval of mutation probabilities (μ<1/*Z* ^*2*^) and for the entire range of possible thresholds *M* required for cooperation to be effective, showing how the signaling systems discussed here are able to secure the necessary coordination needed to achieve a collective benefit, irrespectively of how stringent such conditions are. Indeed, whenever one increases the number of contributions needed to produce a public good (*M*), evolution leads to a higher prevalence of signaling strategies, which in turn leads to greater levels of cooperation. Thus, it is as if *necessity becomes the mother of invention*, as signaling strategies get selected in those situations in which coordinating into collective action is harder to achieve. Additionally, it is important to note that the group size may also play a determinant role in the tendency to cooperate in public goods dilemmas. As shown in [38] for this particular N-person coordination dilemma, as the group size (*N*) approaches the population size (*Z*), we may transform a coordination dynamics characterized by two basins of attraction, into a transformed dynamics in which defectors are always advantageous. Thus, by increasing the group size, we foster a stricter dilemma for strategies that cooperate, independently of their signaling choices. The impact of these two variables—the threshold *M* and group size *N*—is shown in Fig. 3 where we plot the frequency of signaling strategies—i.e., strategies (01|10) and (10|01) as a function of the ratio *M*/*N* and different group sizes.

In this manuscript we have shown that collective signaling and quorum sensing strategies which are sensitive to the majority signal emerge naturally and are robust, allowing populations to coordinate and act appropriately to the externalities imposed by Nature. We have shown that the relevance of these signaling strategies cannot be overlooked, as even when they are not the most abundant in the population, they provide evolutionary barriers that prevent shirkers from dominating in conditions that, in some cases, may put the group survival at jeopardy. This happens even in settings in which response implies producing a public good from which shirkers benefit at no cost. Clearly, the emergence of a signaling system is facilitated when shirking does not translate into cheating but, instead, into abstaining from participating in collective action (see S1 Text). Moreover, whenever harsh conditions become most frequent, evolution selects for a complementary and, arguably, more efficient signaling strategy, in which the absence of signal is used as a costless signal used to foster collective action. Thus, two different signaling systems may emerge depending on the *Nature*’s most prevalent states.

Another limit that is interesting and simplifies the discussion (see S1 Text for additional details) occurs whenever we assume, from the outset, that individuals are constrained to achieve quorum solely in the presence of one particular (costly) signal. If individuals will always defect in the absence of signals, then our strategy space is reduced to 8 instead of the 16-strategy space explored so-far. In this case, the signaling strategy remains always an ESR irrespectively of the value of 0<*λ*<1. Needless to say, whenever Nature’s β states are most frequent, prevalence of signaling may be largely reduced, as shown explicitly in S1 Text. In other words, signaling strategies emerge when collective action is most needed.

Overall, and despite the complexity that collective action relying on quorum signaling may entail, our results clearly show how reliable, robust, and evolutionary viable it is to evolve a signaling system. As a result, it is perhaps no longer so surprising how signaling is a ubiquitous property of the living world.

## Methods

### Signaling Games

Following [38,42–45], we set up a *N*-person signaling game to describe the scenarios advanced before. We assume that Nature can choose between two states, α (with probability *λ*) and β (with probability 1 – *λ*). We assume there exist at most two signals *σ* = {0,1} (*σ* = 0 may also be associated with a *no*-signal), such that all individuals emit either signal *σ* = 0 or signal *σ* = 1. Signals have, *a priori*, no pre-defined meaning, and they may entail a differential cost, say, *σ* = 1 involves an additional cost *c*_*S*_ compared to *σ* = 0, which will have a direct impact in the fitness of individuals emitting such signal. We shall assume the simplest repertoire of strategies concerning the signaling process, that is, individuals act based on the most frequent signal present in the group (flipping a coin in case of a tie). We designate this simple set of strategies by *quorum sensing strategies*.

### Population Dynamics

Let us consider a finite (but of otherwise arbitrary size) well-mixed population of *Z* interacting individuals and assume they reproduce via a birth-death process, implemented here by means of a stochastic update rule [46–49]. At each time step an individual *i* with strategy *S*_*i*_ and fitness Ω*_i_* (obtained in terms of the game payoff Π*_i_* defined below) will be eventually replaced with probability *p* by another random individual *j* adopting a strategy *S*_*j*_ (with fitness Ω*_j_*). The probability *p* increases with the increase in fitness difference between *j* and *i* [48,49], and may be conveniently written in terms of the so-called Fermi distribution (from statistical physics) $p={\left[1+e^{-\gamma \left[\Omega_{j}\left(k\right)-\Omega_{i}\left(k\right)\right]}\right]}^{-1}$, in which *γ* (an inverse temperature in physics) translates here into stochastic errors in the replacement process [48], ultimately defining the intensity of natural selection in the population dynamics: High values of *γ* correspond to very strong selection, whereas for *γ* → 0, selection becomes so weak that evolution proceeds by random drift.

We define an individual strategy *S*_*i*_ as a vector of the form $S_{i}=\left(\sigma_{\alpha },\sigma_{\beta }|A_{0},A_{1}\right)$, where *σ_α_* (*σ_β_*) is the signal emitted by an individual when Nature chooses α (β), and *A*_0_ (*A*_1_) is the action that the individual takes when the majority signal in the group is 0 (1) respectively. The action to a majority signal may be viewed as the simplest possible strategy compatible with quorum sensing—simply act in accordance with the stronger signal. This creates an overall set of *n_S_* = 2^4^ = 16 different strategies, given that we shall consider 2 possibilities of action for each case (see below). One action determines the individual to *cooperate* (***C***) in a Public Goods Game (**PGG**) by contributing a cost *c* to the public good. In line with the game defined in [38], a benefit *b* > *c* will be produced to the extent that at least *M* (where 0 *< M* ≤ *N*) individuals contribute to the **PGG**. We assume the parameterization introduced in [38] and write *b* = *Fkc/N*, with *k* the number of cooperators in the group, and the multiplication factor *F* ≥ 0 a real number which will allow us to describe a variety of scenarios commonly observed in nature. For this game, and unlike the more popular *N*-person Prisoner’s Dilemma, in which unconditional cooperators dominate unconditional defectors whenever *F*>*N*, here even in this case unconditional cooperators face a coordination problem when co-evolving with unconditional defectors [38]. We shall study the evolutionary competition between *cooperators* and *shirkers*. The latter behavior will reflect two different possibilities: in some cases we associate the *shirker* with a conventional *defector* (***D***, or *cheater*), in which case he foregoes the cost while reaping a share of the public good (as long as there are enough *cooperators* in the group); in other cases, the *shirker* behaves as a *loner* (***L***) (see S1 Text) abstaining from contributing to the **PGG**, but also abstaining from reaping the benefits resulting from successful collective action. In summary, when under α, a *shirker* who behaves as a *defector* in a group with *k cooperators* gets $\Pi_{D}\left(k\right)=\frac{Fkc}{N}\theta \left(k-M\right)$ (where the Heaviside step function *θ*(*x*) satisfies *θ*(*x* < 0) = 0 and *θ*(*x* ≥ 0) = 1); a *shirker* who behaves as a *loner* will get 0, whereas a *cooperator* will get Π*_C_*(*k*) = Π*D*(*k*)–*c*, when playing against ***D***s and Π*_C_*(*k*) = *Fcθ*(*k* – *M*) – *c*, when playing against ***L***s. If nature chooses β, the same payoff expressions apply, but now with *F* = 0.

Assuming a well-mixed population, individuals assemble into groups of size *N*, and benefits will accrue to those who *cooperate* or *defect* every time a group contains a number *cooperators* exceeding the threshold *M*. Fitness is, thus, associated with the expected payoff respectively by

$$\Omega_{C}\left(k\right)={\left(\begin{array}{c} Z-1\\ N-1 \end{array}\right)}^{-1}\sum_{j=0}^{N-1}\left(\begin{array}{c} Z-k\\ N-j-1 \end{array}\right)\Pi_{C}\left(j+1\right)$$

and

$$\Omega_{D,L}\left(k\right)={\left(\begin{array}{c} Z-1\\ N-1 \end{array}\right)}^{-1}\sum_{j=0}^{N-1}\left(\begin{array}{c} k\\ j \end{array}\right)\left(\begin{array}{c} Z-k-1\\ N-j-1 \end{array}\right)\Pi_{D,L}\left(j\right)$$

Besides reproduction events, we further assume that with a probability *μ* individuals may mutate to a randomly chosen strategy, freely exploring the space of *n_S_* possible strategies.

### Small Mutation Approximation

A full analysis of the entire configuration space is unfeasible. Hence we adopt the limit *μ→0* (so-called small-mutation limit) [50,51] in which case the analysis of the 16-strategy space becomes tractable. In the absence of mutations, the end states of evolution are inevitably monomorphic, as a result of the stochastic nature of the evolutionary dynamics and update rule. By introducing a small probability of mutation, every time a new mutant appears, the population will either end up wiping out the mutant or witness the fixation of the intruder. Hence, in the small-mutation limit, the mutant will fixate or will become extinct before the occurrence of another mutation and, for this reason, the population will spend all of its time with a maximum of two strategies present simultaneously. This allows one to describe the evolutionary dynamics of our population in terms of a reduced (and embedded) Markov Chain of size *n_S_* [50,51], where each state represents a possible monomorphic end-state of the population associated with a given strategy, and the transitions between states are defined by the fixation probabilities of a single mutant of one strategy in a population of individuals who adopt another strategy. The resulting *stationary distribution* characterizes the average time the population spends in each of these monomorphic states, and can be computed analytically (see below).

### Stationary Distribution

Given the above assumptions, it is now possible to write down the probability to change the number *k* of individuals with a strategy ***U*** (by plus or minus one in each time step) in a population with *Z*–*k* ***V***-strategists: $T^{\pm }\left(k\right)=\frac{Z-k}{Z}\frac{k}{Z-1}{\left[1+e^{-\gamma \left[\Omega_{U}\left(k\right)-\Omega_{V}\left(k\right)\right]}\right]}^{-1}$ [48]. This can be used to compute the fixation probability of a mutant with a strategy ***U*** in a population with *Z-1* ***V***s, given by $\rho_{V,U}={\left(\sum_{Z-1}^{i=0}\prod_{j=1}^{i}\phi_{j}\right)}^{-1}$, where $\phi_{i}=\frac{T^{-}\left(i\right)}{T^{+}\left(i\right)}$ [23,48,52,53]. In the limit of neutral selection (*γ* → 0), *ϕ*_*i*_ becomes independent of the fitness values: *ρ_V,U_* = 1/*Z* [46,48]. Considering a set {1,…,*n_S_*} of different strategies, the fixation probabilities define $n_{s}^{2}$ transition probabilities of the reduced Markov chain, with the associated transition matrix

$$T=\left[\begin{array}{cccc} 1-\eta \left(\rho_{1,2}+\cdots +\rho_{1,n_{S}}\right) & \eta \rho_{1,2} & \cdots & \eta \rho_{1,n_{S}}\\ \eta \rho_{2,1} & 1-\eta \left(\rho_{2,1}+\cdots +\rho_{1,n_{S}}\right) & \cdots & \eta \rho_{2,n_{S}}\\ \cdots & \cdots & \cdots & \cdots \\ \eta \rho_{n_{S},1} & \cdots & \cdots & 1-\eta \left(\rho_{n_{S},1}+\cdots +\rho_{n_{S},n_{S}-1}\right) \end{array}\right]$$ (1)

where *η* = (*n_s_*–1)^–1^ provides the appropriate normalization factor. The normalized eigenvector associated with the eigenvalue 1 of the transpose of *T* provides the stationary distribution described before [50,51]. It is also noteworthy that, as the population spends most of the time in the vicinity of monomorphic states, the fraction of time the population spends in states in which individuals cooperate with its own strategy also corresponds to the fraction of time the population spends in cooperative scenarios. Consequently the stationary distribution obtained from the matrix *T* provides both the relative evolutionary advantage of each strategy, and also the stationary fraction of cooperative acts. Finally, we assume that a strategy *A* is evolutionary robust (ERS) [40,41], if the fixation probability of a single mutant is smaller than neutral fixation, that is, 1/*Z* [46,48]. As shown, this measure offers limited predictive capabilities in what concerns the long-term dynamics and prevalence of each strategy in finite populations, a feature previously discussed in various contexts (see, e.g. [45,50,54–56]). Analogous or poorer predictions are also obtained with other robustness or stability measures. If we add an additional constraint to the above ERS condition, requiring that a single mutant of any other strategy shows a lower fitness than *A*, we obtain the evolutionary stability condition of Refs. [23,46]. The corresponding stability analysis would yield even worse predictions, in the sense that parameter regions in which signaling strategies would be both long-term prevalent and stable would be reduced. Likewise, we obtain the same results shown in Fig. 1 if an evolutionary stable strategy is defined as a strategy *A* which, if invaded by an arbitrary mutant *B* with probability *ρ*_*A*,*B*_>1/*Z*, is able to counter invade with probability *ρ*_*B*,*A*_, larger than *ρ*_*A*,*B*_ [55]. This said, and irrespective of the definition of evolutionary stability adopted in finite populations, the overall dynamical picture and conclusions described here still hold, being thus independent of the particular measures of robustness and stability considered.

## Supporting Information

S1 Text Supporting Text (containing 5 additional figures) provides additional details concerning the methodology adopted (Section S1) and investigates *i)* the evolutionary dynamics in the limit cases when Nature only chooses *A* and *B* states (Sections S2 and S3); *ii)* the evolution of cheap forms of quorum depending on Nature’s state (Section S4); *iii)* the evolution of signaling strategies when individuals are constrained to achieve quorum solely in the presence of one particular (costly) signal (Section S5); and *iv)* the emergence of signaling in the evolutionary dynamics of *Cooperators* and *Loners* (Section S6). (PDF)
