# Supplementary material for: Co-evolutionary Dynamics of Collective Action with Signaling for a Quorum
Source: PLoS Comput Biol. 2015 Feb 23;11(2):e1004101. doi: 10.1371/journal.pcbi.1004101 (PMC4338077; doi:10.1371/journal.pcbi.1004101)
Supplement: S1 Text — (PDF) [file pcbi.1004101.s001.pdf]

SUPPORTING INFORMATION

Co-evolutionary Dynamics of Collective Action with Signaling for a Quorum

Jorge M. Pacheco[1,2,3], Vítor V. Vasconcelos[3,4,5], Francisco C. Santos[5,3] & Brian Skyrms[6]

[1] Centro de Biologia Molecular e Ambiental, Universidade do Minho, 4710 - 057 Braga, Portugal

[2] Departamento de Matemática e Aplicações, Universidade do Minho, 4710 - 057 Braga, Portugal

[3] ATP-Group, CMAF, Instituto para a Investigação Interdisciplinar, P-1649-003 Lisboa, Portugal

[4] Centro de Física da Universidade do Minho, 4710 - 057 Braga, Portugal

[5] INESC-ID and Instituto Superior Técnico, Universidade de Lisboa, IST-Taguspark, 2744-016 Porto Salvo, Portugal

[6] Logic and Philosophy of Science, School of Social Sciences, University of California at Irvine, Irvine, CA 92612, USA

S1. States of Nature and Game Payoffs

We define an individual strategy S_i as a vector of the form $S_i = (\sigma_\alpha, \sigma_\beta \mid A_0, A_1)$, where $\sigma_\alpha (\sigma_\beta)$ is the signal emitted by an individual when Nature chooses α (β), states which can be metaphorically associated with Abundance (β) and Starvation (α), whenever one considers microbial organisms as potential candidates. $A_0 (A_1)$ is the action that the individual takes when the majority signal in the group is 0 (1). This creates an overall set of $n_S = 2^4 = 16$ different strategies, given that we shall consider 2 actions for each case. One action determines the individual to *cooperate* (***C***) in a Public Goods Game (**PGG**) by contributing a cost *c* to the public good [1]. A benefit $b = Fn_C c / N$ will be produced to the extent that at least *M* (where 0 < *M* ≤ *N*) individuals contribute to the **PGG**. n_C is the number of cooperators in the group, and the multiplication factor $F \geq 0$ is a real number. We shall study the evolutionary competition between *cooperators* and *shirkers*. S*hirkers* are either *defectors* (***D***, or *cheaters*), in which case they forego the cost while ripping a share of the public good (as long as there are enough *cooperators* in the group), or *loners* (***L***), abstaining from contributing to the **PGG** but also abstaining from ripping the benefits resulting from successful collective action. In summary, we write the game payoffs of ***D***s and ***L***s respectively as $\Pi_D(k) = \frac{F k c}{N} \theta(k - M)$ and $\Pi_L(k) = 0$ (*k* is the number of *cooperators* in the group and the Heaviside step function $\theta(x)$ satisfies $\theta(x < 0) = 0$ and $\theta(x \geq 0) = 1$). *Cooperators*, in turn, get $\Pi_C(k) = \Pi_D(k) - c$, when playing against ***D***s, and $\Pi_C(k) = F c \theta(k - M) - c$, when playing against ***L***s.

The payoffs above are realized whenever Nature chooses α, associated with the probability λ. Whenever Nature chooses β, the same payoff expressions are valid, but now with $F = 0$.

S2. Nature only chooses β

When populations only face situations of β (λ = 0.0), no quorum sense signaling system is required and everyone will be better off by reproducing individually. In this situation cooperation will remain costly while no benefits accrue to those who cooperate: Nature does not reward cooperation towards the public good under β. Given the alternatives of emitting a costly signal (signal 1) or emitting no signal (signal 0), and given the fact that cooperation is of no use in this case, the best case scenario will correspond to individuals emitting no signal under *A* and *shirking* under majority of "signal 0". Since there is no selection under α, what they do under "signal 1" should be irrelevant. In other words, in the best case scenario the population should spend all of its time in configurations of the type (*0|0*), where the placeholder "*" means here any value. As a result, one expects that strategies (00|00), (10|00), (00|01) and (10|01) take 25% of the total time each. Results are shown in Figure S1 and nicely confirm the expectations.

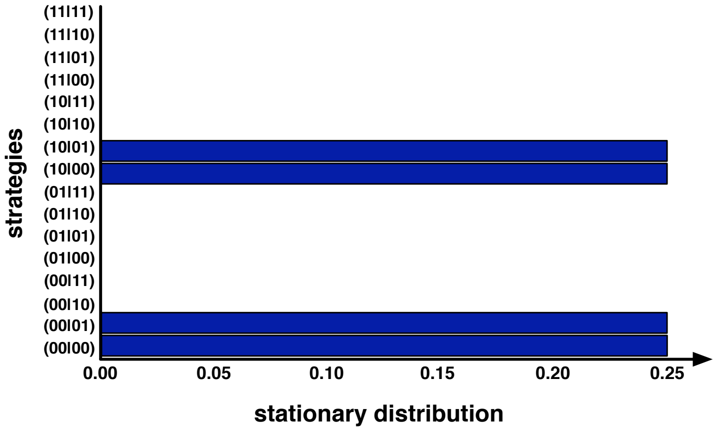

Figure S1. Strategy prevalence when Nature only chooses β (λ = 0.0). In this case, the population evolves strategies (listed along the Y-axis) where they should learn to defect when no signal is emitted. This encompasses 4 out of the 16 possible strategies, which indeed share 25% of the total time (listed on the X-axis). The reason why these strategies are degenerate has to do with the fact that no selection pressure exists to discriminate amongst them. Model parameter values: Z=200, N=9, M=5, c=0.5, c_S=0.1, F=10, γ=5.

S3. Nature only chooses α

Let us now assume that Nature only chooses α (λ = 1.0). Under α, continuity of the species may rely on cooperation. Thus cooperation remains costly, but now collective cooperative action will entail a public good. Under α, Nature may reward signaling. However, given the fact that there is no pressure to distinguish between α and β, individuals will be better off by evolving to cooperate in the absence of any (costly) signal. Hence, and similar to the situation studied before, strategies (0⋆|1⋆) realize the best case scenario in this case. The results, which fully corroborate this prediction, are shown in Figure S2.

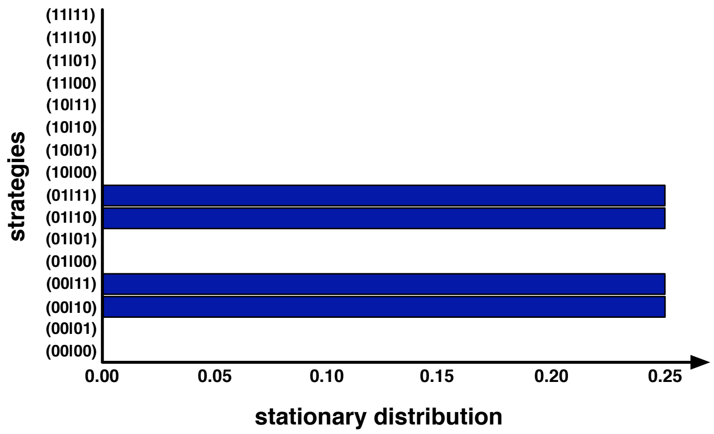

Figure S2. Strategy prevalence when Nature only chooses α (λ = 1.0). In this case, individuals should evolve to cooperate when no signal is emitted. This encompasses 4 out of the 16 possible strategies, which indeed share 25% of the total time (listed on the X-axis). As in section S2, the reason why these strategies are degenerate has to do with the fact that no selection pressure exists to discriminate amongst them. Model parameter values: Z=200, N=9, M=5, c=0.5, c_S=0.1, F=10, γ=5.

Importantly, the results discussed above do not change irrespective of whether there is a game between ***C***s and ***D***s or between ***C***s and ***L***s, that is, whether shirkers behave as ***D***s or ***L***s.

S4. Evolution of cheap quorum

Whenever Nature chooses α more often than β (large λ) a second signaling strategy may also become dominant, as a natural extension of the scenario already discussed before for the case of λ = 1. Evolution selects for a strategy (01|10), a discriminative strategy in which

individuals react cooperatively in the absence of a costly signal, and defect whenever a costly signal is present. In other words, individuals use a collective analog of cheap talk (and a secret, collective handshake) to coordinate in case of α (most cases), whereas in case of β they signal "1", which in turn leads others to avoid paying the cost of cooperation. This strategy — which corresponds to the complement of the signaling strategy, (10|01), discussed above — benefits from the fact that *i)* collective action is achieved solely when needed and *ii)* costly signals are seldom used. The prevalence of this type of strategy may also explain why costly signals may prevail in periods when Nature chooses β, in particular, when (apparently) they are not needed. Thus, their existence, even if costly, allows the assignment of a meaning to the absence of signals, which is then used profitably by the species in times of need. This said, and in contrast with (10|01) this cheap quorum is not evolutionary robust (ERS), being disadvantageous with respect to (10|01) in most cases, as shown in Figure 1.

S5. Reducing the space of possible strategies

We have been assuming that individuals may react to both signals 0 and 1, and no *a priori* meaning to the signals were assigned. However, we may argue that, in some cases, individuals may be constrained to achieve quorum solely in the presence of one particular (costly) signal. If individuals will always defect in the absence of signals, then our strategy space is reduced to 8 of the 16-strategies set explored above including one single signaling strategy (10|01), with the following evolutionary outcome. The signaling strategy remains always an ERS irrespectively of the value $0<\lambda<1$. Needless to say, depending if β states are dominant, their prevalence may be largely reduced, as shown in Figure S3.

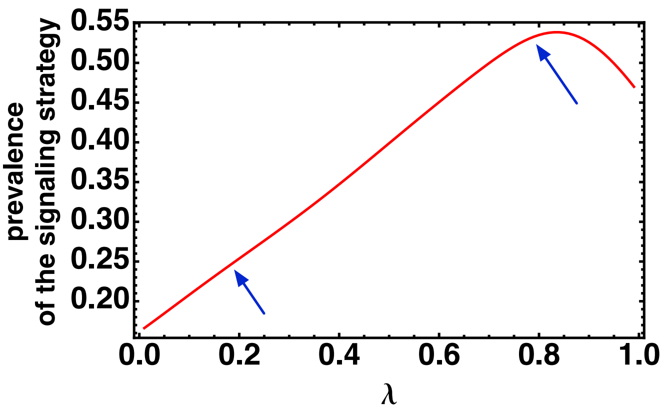

Figure S3. Signaling prevalence for different state probabilities under variable A**.** The red curve shows prevalence of the signaling strategy (10|01) (directly obtained from the stationary distribution, see Methods) as a function of the probability of Nature choosing α, λ. The arrows indicate the values of λ at which the dynamical analysis of Figure S4 (see below) is carried out. Model parameters: Z=100, N=9, M=5, c=0.3, c_s=0.06, γ=0.5, F=10.

Figures S4a and S4b show the transition probabilities and respective prevalence of each strategy for two representative values of λ (0.2 and 0.8) indicated by arrows in Figure S3.

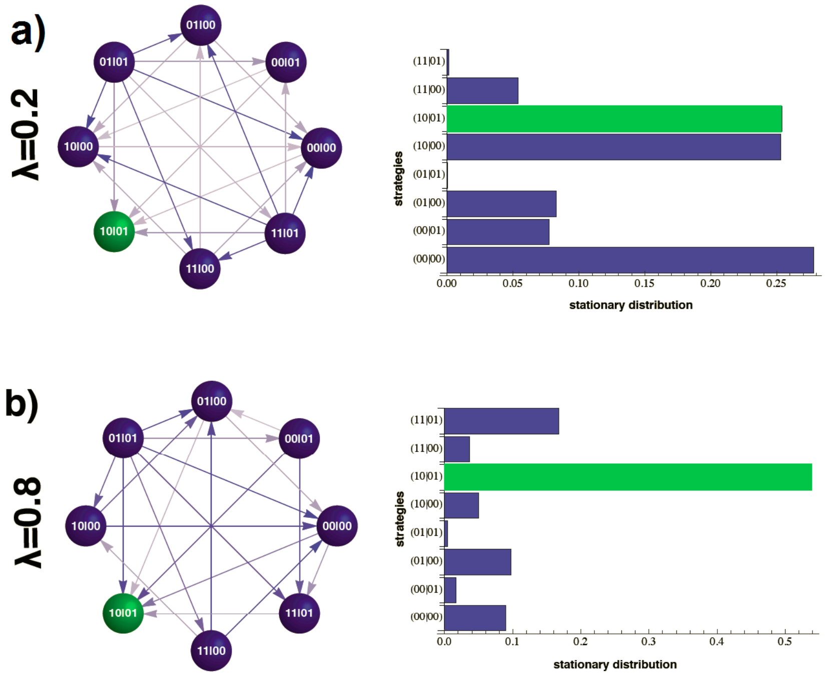

Figure S4. Dynamics. The bar plot shows the fraction of time the population spends in each monomorphic configuration. We use the same notation and convention as in Figure 2 of main text. The graph represents all strategies, one at each node, with edges representing transitions between strategies above neutral drift, with thickness proportional to intensity. The green node and bar denote the signaling strategy. Model parameters: Z=100, N=9, M=5, c=0.3, c_s=0.06, γ=0.5, F=10.

S6. Playing against Loners

As stated in the main text, there are situations in which shirkers may act as *Loners* instead of *Defectors*. In Figure S5 we show the results of the same analysis carried in the context of Figure 1 of the main text, regarding the prevalence of the signaling strategy (10|01) in the evolutionary dynamics of *Cooperators* and *Loners*.

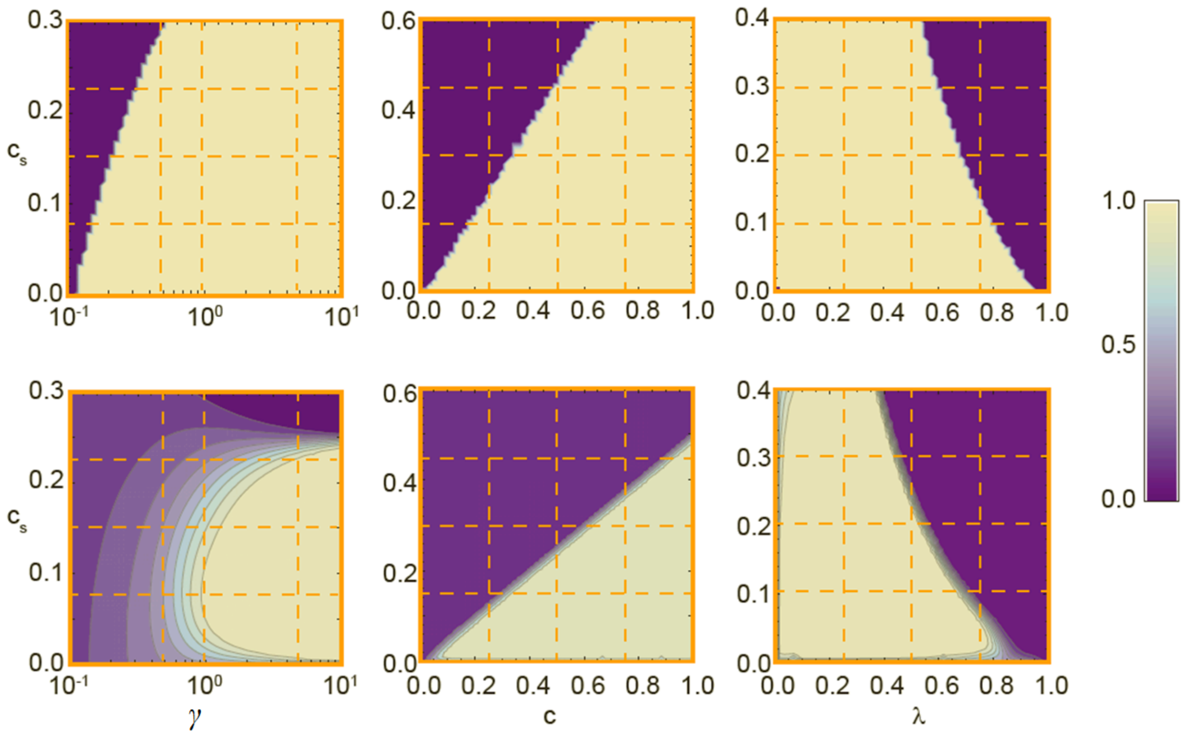

Figure S5. Evolutionary Robustness and Prevalence of signaling strategy (10|01) **when playing with Loners.** We use here the same notation as in Figure 1 of main text. Thus, in the **top** contour plots, we depict the regions of model parameters in which the signaling strategy (10|01) is evolutionary robust (ERS) (bright areas, corresponding to 1 on the scale). The **bottom** panels show, for the same parameter space, the prevalence of (fraction of time) the population in a configuration in which all individuals adopt the signaling strategy (10|01). The remaining parameters (and also those plotted whenever not varied) are Z=100, N=9, M=5, c=0.5, γ=5, F=10, λ = 0.5. Similar to what was observed for defectors, when shirkers act as loners a signaling system also emerges and, in general, for a wider range of values of the model parameters.

Similarly to Figure 1, a signaling system also emerges in this case and, in general, for a wider range of values of the model parameters when compared to the game with *Defectors*. Comparison of top and bottom panels once again shows the existence of parameter regions in which (10|01) is an ERS and yet the population almost never adopts this strategy and vice-versa (see main text for details). In Figure S5, Loners are those that remain aloof with respect

to the public good at stake, leaving the collective benefits, whenever achieved, to be shared amongst those that participate in the PGG. There may be situations, however, in which the share that each one gets is still $1/N$ of the public good, and not $1/n_C$. The results shown in Figure S5 remain very much unaffected by this change.

References

1. Pacheco JM, Santos FC, Souza MO, Skyrms B (2009) Evolutionary dynamics of collective action in N-person stag hunt dilemmas. Proc R Soc B 276 (1655): 315-321.
